# Clinical outcome of skin yaws lesions after treatment with benzathinebenzylpenicillin in a pygmy population in Lobaye, Central African Republic

**DOI:** 10.1186/1756-0500-4-543

**Published:** 2011-12-15

**Authors:** Alexandre Manirakiza, Susana Vilas Boas, Narcisse Beyam, Germain Zadanga, François Xavier Konamna, Siméon P Njuimo, Rémi Laganier

**Affiliations:** 1 Institut Pasteur de Bangui, PO Box 923, Bangui, Central African Republic; 2 Poste de santé de Mongoumba, Bangui, Central African Republic; 3 United Nations Population Fund, Bangui, Central African Republic

**Keywords:** Yaws, Treatment, Central African Republic

## Abstract

**Background:**

Yaws is a bacterial skin and bone infectious disease caused by *Treponema pallidum pertenue*. It is endemic, particularly among pygmies in Central African Republic. To assess the clinical cure rate after treatment with benzathinepenicillin in this population, we conducted a cohort survey of 243 patients in the Lobaye region.

**Findings and conclusion:**

The rate of healing of lesions after 5 months was 95.9%. This relatively satisfactory level of therapeutic response implies that yaws could be controlled in the Central African Republic. Thus, reinforcement of the management of new cases and of contacts is suggested.

## Background

Yaws is a skin and bone non-venereal treponematosis caused by *Treponema pallidum *subsp. *pertenue*. It is not considered a neglected disease, but a forgotten one [1,2]. Most infected people reside in warm, humid tropical areas, in communities with lack of hygiene [3]. The usual means of transmission of yaws is close bodily contact with a patient with infectious lesions [4]. The clinical features are classified in stages [1,5]. During the primary stage, a lesion called the 'mother yaw' occurs as a papule, which develops after 2-4 weeks of incubation at the site of *Treponema *inoculation and enlarges before it ulcerates. Spontaneous resolution occurs after almost 6 months, and the initial lesion heals. The secondary stage is characterized by widespread smaller skin papules, the 'daughter yaws'. After a variable latency, which can last several years, a late stage develops in 10% of patients, which consists of skin ulceration, gumma formation and destruction of bones and cartilage. Painful palmoplantar hyperkeratosis and keratoderma are also observed during this third stage.

Basically, the diagnosis of an infection by Treponema pertenue is based on three criteria,(i) the emergence of the disease in an endemic region, (ii) clinically typical papilloma lesions, and (iii) seroactivity in a treponemal antigen test [6]. The operational definition of a case of yaws is "any person who lives in an endemic area and presents with one or more of the following signs: painless ulcer with scab, papilloma, palmar/plantar hyperkeratosis (thickening)" [2].

Clinical diagnosis is reliable with minimal training of health staff. In the field, diagnosis is based mainly on clinical findings and epidemiology. The diagnostic terms suggestive of active yaws proposed for use in mass campaigns [7-9] are (i) initial lesions of nongenital chancre, painless, non-tender papule with a raised margin; (ii) multiple papillomata; (iii) plantar and palmar papillomata; (iv) hyperkeratosis and (v) gummata, ulcers and gangosa.

The elimination and ultimate eradication of the infection require strategies targeted against the factors that favour its transmission. Thus, control activities must be considered in mass treatment campaigns to eliminate the source of infection, with improved environmental and individual hygiene [10]. Benzathinebenzylpenicillin is the currently recommended antibiotic for use against yaws [11].

Mass campaigns carried out in yaws endemic areas since the 1950's gave rise to the possibility of eradication of this infection [11,12]. Recently, however, resurgence of yaws has been reported [13-15], due to curtailment of control activities, which has allowed the reservoir of untreated yaws to grow unchecked.

Yaws is endemic in central Africa [16]. In the Central African Republic (CAR), pygmies are the most widely infected segment of the population [6]. In a serological survey performed between November 1978 and March 1979 in the pygmy population of southwest CAR (Lobaye and Sangha), the prevalence of clinically diagnosed yaws was 50%, with positive serology rates of 86% in children and 95% in adults [17]. In the same area, Cirera et al. showed that 78.5% of pygmy children had sera positive for *Treponema*, suggesting massive infestation with *T. pertenue *[18]. Mass treatment campaigns were carried out in those regions between 1977 and 1980 (CAR Health Ministry, unpublished data). In 1992, a resurgence of yaws was reported in this area [15], but no further control activities were conducted.

Treatment of yaws with benzathinebenzylpenicillin is included in CAR Ministry of Health guidelines; however, the outcome of patients treated with this antibiotic has never been assessed. The aim of this study was to evaluate the clinical evolution of yaws skin lesions after administration of benzathinebenzylpenicillin in a pygmy population.

## Methods

### Study area

**Figure 1:**
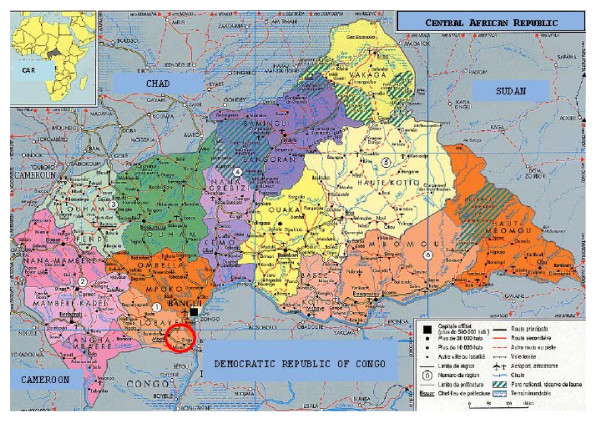
**Study location, Mongoumba, Central African Republic**.

A treatment campaign against yaws was conducted in Mongoumba (Figure 1), a district in the Lobaye region in southwest CAR close to the borders with the Democratic Republic of the Congo and the Congo. The climate is equatorial. The total population is estimated at 21 235 inhabitants, 15% (n = 3089) of whom are pygmies. In this area, there are two health centres: the Santé Saint Georges, which is a private health centre run by Comboni Missionaries whose aim is to improve the social integration of pygmies; and the Mongoumba Health Centre, which is a public institution of the CAR Ministry of Health.

Staff of Santé Saint Georges visit pygmies in their settlements, dispersed throughout the forest, to advise them on hygiene and health services. Patients are invited to present themselves to either the Santé Saint Georges or the Mongoumba health centre.

### Case diagnosis

Between November 2007 and March 2008, a cohort of pigmies presenting with skin lesions was constituted. The inclusion criteria were skin lesions clinically suggestive of yaws or any other non-traumatic skin lesions that could not be classified but which scored positive on serological analysis with both the venereal disease research laboratory (VDRL) test and the *T. pallidum *haemagglutination (TPHA) test.

All 254 pygmies in 18 settlements in the Mongoumba region with these types of skin lesion were recruited. Of these, 191 had skin lesions clinically suggestive of yaws, and 63 had other skin lesions. Assuming that *Treponema *serology was likely to be positive in people with skin lesions clinically suggestive of yaws [17,18], we did not collect blood samples for serological diagnosis in these cases; however, a 5-ml venous blood sample was collected from each of the 63 pygmies with atypical skin lesions and tested at the Institut Pasteur de Bangui. Both tests were positive in 52 patients, three were positive with TPHA and negative with VDRL, and eight samples were negative with both tests. The patients positive in both tests were considered to have active yaws. Thus, a cohort of 243 patients was constituted to assess scarring of skin lesions after injection of one dose of benzathinebenzylpenicillin.

### Treatment and follow-up

During April 2008, a single dose of benzathinebenzylpenicillin was administered intramuscularly to each of the 243 patients in our study cohort and to contacts not presenting any skin lesions. The doses administered were 2.4 M units for adults, 1.2 M units for children and 0.6 M units for infants. Some pygmies were absent during this treatment campaign. Hence, a total of 2456 pygmies in the Mongoumba area (79.5% or 2456/3089) were treated during the 6-month campaign. Follow-up of those pigmies with skin lesions was conducted at the Santé Saint Georges health centre, with no follow-up in the settlements. Clinical outcome (total scarring of skin lesions) was assessed 2-5 months later (July-September 2008).

### Ethical approval and consent to participate

Because there was no national ethical committee in the CAR during the period in which this survey was conducted, the project was approved by the expert committee for drug policy and the Ministry of Health in the CAR. The aim of the survey and all the procedures (clinical examination, probable collection of blood samples for laboratory investigation of yaws, injection of the antibiotic into the gluteal muscle, use of photographs for possible publication and duration of follow-up) were explained in the native Aka language during meetings organized by our study team. Oral consent was collected from all participants, none of whom was literate.

## Findings

**Figure 2:**
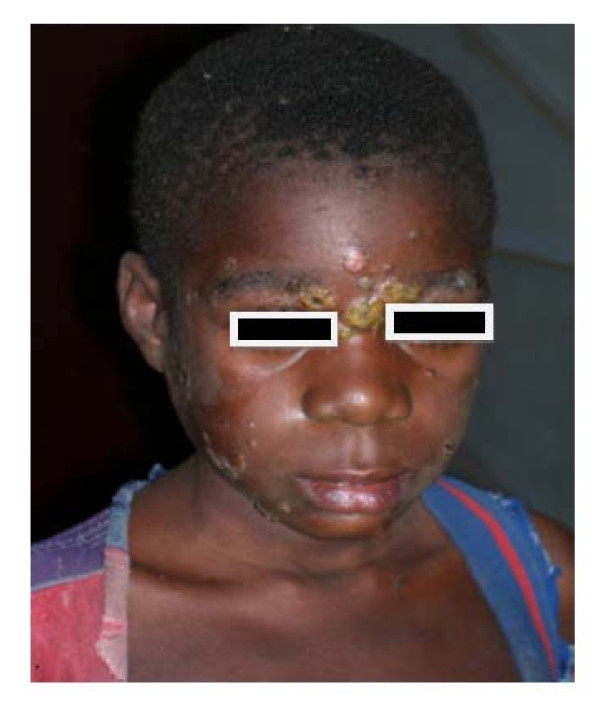
Active yaws lesions, Mongoumba, Central African Republic, December 2007 (photograph by S.V. Boas).

**Figure 3:**
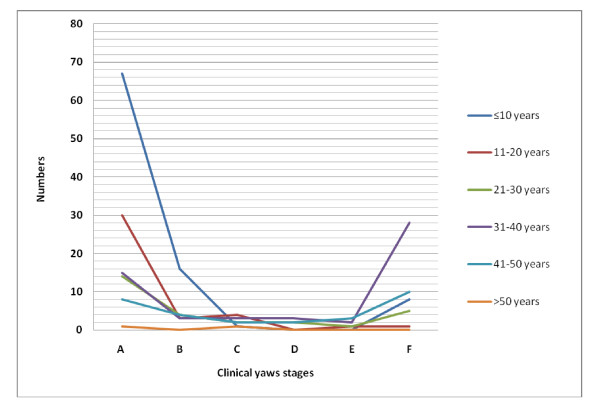
**Age distribution by stage of yaws in 243 patients, Central African Republic, December 2007-September 2008**.

In this study, we observed no lost of follow-up. The mean age of patients was 16 years (median, 20 years) and the male: female ratio was 1.15. Initial yaws skin lesions were found in 135 (55.6%) cases, multiple papillomata (Figure 2) in 12.3%, plantar and palmar papillomata in 5.3%, hyperkeratosis in 2.5% and ulcers in 2.9%; the remaining cases, classified as atypical lesions, represented 21.4%. There was no statistically significant difference in the distribution of clinical stages by sex (*p *= 0.6). Initial yaws lesions were more frequent among children < 17 years (74.5% or 82/110) than in other age categories (*p *< 0.0001) (Figure 3).

**Figure 4:**
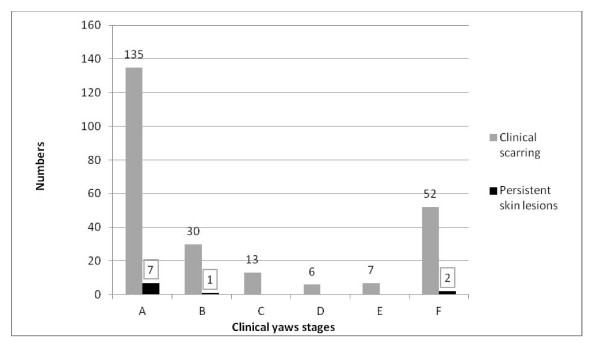
**Clinical healing after treatment of yaws in 243 patients, Central African Republic, December 2007-September 2008**.

Overall, 95.9% (233/243) of the skin lesions had scarred during the period before outcome assessment (Figure 4); 10 patients presented with persistent skin lesions at the end of assessment, all located on the feet and complicated by ulcers. These persistent lesions had been classified at baseline as initial lesions (5.2% or 7/135), multiple papillomata (3.3% or 1/30) and atypical skin lesions (3.8% or 2/52). Persistent lesions were followed up and dressed regularly at the Santé Saint Georges health centre.

The additional file 1 (as Excel xls file) provide details on data of this study.

## Discussion

There is a suspected emergence of *Treponema pertenue *resistance to benzathinebenzylpenicillin [19]. Hence, it was essential to assess clinical evolution of skin lesions of yaws after treatment with benzathinebenzylpenicillin. Our findings show that relatively few skin lesions persisted 5 months after administration of benzathinebenzylpenicillin in this study cohort. The persistent lesions might be treatment failures due to concurrent factors such as other infecting agents and microtrauma. All the persistent lesions were located on the feet, which are highly exposed because pygmies rarely wear shoes and their economy is based on hunting in the equatorial forest. It is possible that *T. pallidum pertenue *developed resistance to benzathinebenzylpenicillin in these cases, as reported by Backhouse in Papua New Guinea [19,20].

A potential limitation of this study is that serological tests were not performed systematically in all patients to support a diagnosis of yaws, and clinical diagnosis might have led to overestimation of the number of cases. In this endemic context, however, skin lesions are highly predictive of yaws [17]. Moreover, we found 82.5% positive serology with both VDRL and TPHA in patients with atypical lesions clinically suggestive of yaws. A second potential limitation is that the outcome was not assessed from VDRL titres after treatment but only from healing of skin lesions. Use of VDLR titres could be justified in chronic infections involving the bones [19], because of low bone penetration of penicillin [21]. Scarring of skin lesions can be attributed to killing of the microbes by benzathinebenzylpenicillin a few months after its administration to patients, even if spontaneous resolution of some primary lesions occurred. Moreover, no new skin lesions suggestive of yaws were registered at Santé Saint Georges or in the pygmy settlements at the time we measured clinical outcome. Therefore, the mass campaign with benzathinebenzylpenicillin prevented any new infections, suggesting its efficacy.

The high proportion (26.5%) of initial lesions in people aged over 17 years is surprising. In this region, a diagnosis of yaws can be differentiated from primary lesions of tropical ulcers [22,23], hence, usefulness of serological tests to overcome this difficulty with diagnosis.

Although the coverage rate of our mass treatment campaign was almost 80%, there could be latent yaws lesions in the untreated pygmies, constituting a source of resurgence of active yaws lesions in Mongoumba. The scarring rate observed in this cohort survey indicates that mass campaigns could eradicate this infectious disease. It is, however, not easy to give health advice to pygmies who live in the forest. Continued interruption of transmission and eradication depend on the availability of personnel and environmental sanitation.

## Conclusion

This survey showed a high rate of efficacy of benzathinebenzylpenicillin in healing yaws. Our findings also show that yaws is presumably still highly prevalent in this area, especially in the pygmy population, due to their poor personal and environmental hygiene. As spread of this infection to other contact groups in the country is possible, ongoing activities to improve social conditions and surveillance and prompt treatment of new yaws cases and all contacts are necessary in the endemic areas of CAR. The CAR health Ministry should introduce yaws control activities in the primary health care and further serological studies to assess the efficacy of benzathinebenzylpenicillin are also needed, with assessment of the bone stage of yaws.

## Competing interests

The authors declare that they have no competing interests.

## Authors' contributions

AM and SVB conceived the study, did the data management and drafted the paper. The field study was conducted by AM, SVB, NB, GZ, FXK and SPN. RL participated in laboratory analysis and interpretation. AM and SVB prepared this draft. All authors read and approved the final manuscript.

## Supplementary Material

Additional file 1 **Clinical yaws stage: A, initial lesions; B, multiple papillomata; C, plantar and palmar papillomata; D, hyperkeratosis; E, gummata, ulcers or gangosa; F, atypical skin lesions**. Click here for file
